# Traumatic Upper Extremity Nerve Lesions in Children: High-Resolution Nerve Ultrasound Can Improve Surgical Outcome

**DOI:** 10.1055/a-2716-2279

**Published:** 2025-10-24

**Authors:** Natalie Winter, Alexander Grimm, Johannes Heinzel, Julia Wittlinger, Josua Kegele, Justus Lieber, Cristian Urla, Martin Ulrich Schuhmann, Helene Hurth

**Affiliations:** 1 Department of Epileptology, Center for Neurology, Eberhard Karls University, Tuebingen, Germany; 2 Hertie Institute for Clinical Brain Research, Eberhard Karls University, Tuebingen, Germany; 3 Department of Hand-, Plastic, Reconstructive and Burn Surgery, BG Klinik Tuebingen, Eberhard Karls University, Tuebingen, Germany; 4 Department of Pediatrics V, Pediatric Surgery and Pediatric Urology, Eberhard Karls University, Tuebingen, Germany; 5 Department of Neurosurgery and Neurotechnology, Section of Pediatric Neurosurgery, Eberhard Karls University, Tuebingen, Germany

**Keywords:** nerve injury, high-resolution ultrasound, nerve trauma, outcome predictors, nerve repair

## Abstract

**Background:**

Peripheral nerve injuries may accompany traumatic extremity injuries and are associated with significant morbidity. Diagnostic options are limited in young children since compliance might be restricted. High-resolution ultrasound (HRUS) is a promising technique to close the diagnostic gap, but clear recommendations are lacking. This study evaluates clinical outcomes after conservative versus surgical management, considering HRUS findings in pediatric patients with upper extremity peripheral nerve injuries.

**Methods:**

We retrospectively analyzed our pediatric neurosurgery database from August 2008 to December 2022 including patients < 18 years with traumatic upper extremity nerve injury and excluding obstetrical brachial plexus injury. Systematic HRUS examinations were implemented from 2016 onwards. Clinical, intraoperative, sonographic and electrophysiological findings were assessed.

**Results:**

A total of 73 nerve injuries in 67 patients (median age = 7.0 years) were analyzed. The most frequently affected nerves were the ulnar (49.3%), radial (21.9%), and median nerve (19.2%). At initial presentation, 47.9% underwent electrophysiology and 67.1% received HRUS. Surgery was performed in 49.3% at a median of 4 months posttrauma, whereas 50.7% were managed conservatively. Patients undergoing surgery initially had more severe sensory and motor deficits (χ ^2^ = 3.98, *p* = 0.046), but final outcomes showed no significant difference in nerve function (median follow-up = 6.0 months). Binary logistic regression identified age (odds ratio [OR] = 1.3, *p* = 0.028), HRUS (OR = 10.6, *p* = 0.035), and injured nerve (OR = 3.1, *p* = 0.022) as independent outcome predictors.

**Interpretation:**

Good functional recovery in pediatric patients with peripheral nerve injury was demonstrated. HRUS-guided treatment and age < 9 years were independent predictors of favorable outcome. These findings support HRUS as a valuable, noninvasive tool for guiding pediatric nerve injury management.

## Introduction

Peripheral nerve lesions are injuries which often carry lifelong implications for the affected patients, given the severe sensory and motor deficits, which persist even following adequate treatment. 1 Reports regarding the incidence of pediatric nerve lesions range between 5.7 and 15% of all nerve trauma patients in specialized centers, 2 3 but overall literature is sparce on this subject. Peripheral nerve lesions in children are most often caused by lacerations and cutting injuries or occur following fractures, 2 i.e., supracondylar humerus fracturs that are in 10 to 20% accompanied by lesions of the median, ulnar or radial nerve. 4 5 6 7 Diagnosis and treatment are based on clinical examinations, but their value is often reduced due to the limited ability of small children to show voluntary movements. In addition, imaging methods such as magnetic resonance imaging (MRI) and nerve ultrasound as well as electrophysiological evaluations can contribute valuable insights in such cases and help to make the right diagnosis and treatment choice, respectively. 8 9 While MRI scans regularly require anesthesia or sedation of the affected children to guarantee an artifact-free examination, 10 nerve ultrasound is easily accessible and inexpensive method that allows for precise evaluation of peripheral nerves in the context of suspected trauma. 11 12 13 Although electromyography is the gold standard method to evaluate and monitor peripheral nerve lesions, the pain associated with this examination due to the required intramuscular placement of needle electrodes limits its use in children. 14 15 16 Nevertheless, nerve conduction studies and electromyography are valuable diagnostic measures for the evaluation of pediatric patients with peripheral nerve injuries. While it is undisputed that a timely diagnosis is one of the keystones of adequate treatment, the impact of electrophysiological and ultrasonographic evaluations have yet been rarely investigated in children with peripheral nerve lesions. 17

In our institution we established systematic high-resolution ultrasound (HRUS) of the affected nerve for all pediatric patients with peripheral nerve injuries from 2016 onwards and incorporated the findings into the interdisciplinary decision-making regarding whether to pursue operative or conservative treatment. This work analyzes all pediatric patients with peripheral nerve injuries of the upper extremities treated at our institution between August 2008 and December 2022. We further aimed to investigate the potential effects of HRUS on treatment choice and functional recovery in these patients by dichotomizing patients in groups with- and without HRUS.

## Methods

All patients with traumatic peripheral nerve injuries of the upper extremity, including the brachial plexus, treated at our tertiary care university hospital between August 2008 and December 2022 were included in the analysis. Exclusion criteria were traumatic birth injuries of the brachial plexus and primary surgical treatment of a nerve lesion at another institution.

The primary objective of this study was to evaluate the outcomes of pediatric patients with upper extremity peripheral nerve injuries. Starting in 2016, we systematically performed HRUS examinations of the injured nerves on all patients, and the sonographic findings were integrated into any therapeutic decision-making. A secondary objective was to compare sonographic and intraoperative findings, as well as clinical outcomes prior and after HRUS-guided therapy.

We analyzed patient charts for the following clinical data: mechanism of trauma; injured nerve; when available, HRUS, neurographic, and electromyography (EMG) findings at initial presentation and follow-up; motor and sensory function, pain intensity at first presentation and final follow-up; treatment type (conservative or surgical); and, in surgical cases, the type of surgical procedure and intraoperative findings.

Muscle strength was assessed using the Medical Research Council (MRC) scale, 18 ranging from grade M0 (no activity) to grade M5 (full strength). If more than one muscle was affected, the lowest MRC grade was used. Pain intensity was measured using the verbal rating scale and categorized as no pain, mild pain, moderate pain, or severe pain. 19 20 21 Based on reported pain levels and motor and sensory function, disability was classified as high (M0–1, hand anesthesia, or severe pain), medium (M2–3 or moderate pain), low (M4, hypesthesia or mild pain), or none (normal motor and sensory function, no pain).

Patients were recruited from the Section of Pediatric Neurosurgery database and clinically examined by a pediatric neurosurgeon and/or a neurologist. Surgery was indicated based on the clinical condition, recovery status after 3–6 months, and electrophysiological findings, if available. When high-resolution ultrasound (HRUS) was available, surgery was performed in cases of absent clinical recovery and pathological sonographic findings but not when sonography was unremarkable. HSUS was performed with a Canon Aplio i800 Ultrasound System (CANON MEDICAL SYSTEMS CORPORATION) using a 24 MHz linear probe. All sonographic, neurographic, and electromyographic examinations were conducted at the Department of Neurology. EMG was performed under mild analgosedation for patients younger than 7 years.

### Statistic

For statistical analysis and figure creation, we used IBM SPSS Statistics 28 (IBM Corporation, Armonk, New York, United States) and Microsoft Excel 16.88 (Microsoft Corporation, Redmond, Washington, United States). The Shapiro–Wilk test was employed to assess the normal distribution of metric variables. Data were compared using *t* -tests or the Mann–Whitney U test as appropriate. Nominally scaled data were analyzed using the chi-square test. Disability levels were determined based on motor function, sensory function, and pain, and classified as high, moderate, low, or no disability. For subgroup analyses, these categories were dichotomized into high versus moderate/low disability groups. To identify independent predictors of unfavorable clinical outcomes, variables found to be statistically significant in univariable analyses were included in a binary logistic regression model. A *p* -value of <0.05 was considered statistically significant (95% confidence interval).

### Ethics Approval

All diagnostic and surgical procedures were performed in accordance with the ethical standards of the institutional research committee and the Declaration of Helsinki from 1964 (as amended over time). Ethical approval for this study was obtained from the Ethics Commission at the Medical Faculty of Eberhard Karls University Tuebingen (007/2020BO2). None of the data acquired as part of our in-house routine protocol required additional informed patient consent.

## Results

A total of 73 nerve injuries in 67 patients were included in the analysis. The median patient age was 7.0 years (range: 0–17 years). A male predominance was observed, with 67.2% ( *n* = 45) male and 32.8% ( *n* = 22) female cases. The most frequently injured nerve was the ulnar nerve, accounting for 49.3% ( *n* = 36) of cases, followed by the radial nerve (21.9%, *n* = 16) and the median nerve (19.2%, *n* = 14). Injuries to the brachial plexus were seen in 8.2% ( *n* = 6) of patients. The most common mechanism of injury was bone fractures (76.7%, *n* = 56), with humeral fractures being the most frequent (63.0%, *n* = 46). A summary of the demographic data are provided in Table 1 .

**Table 1:** Demographic data and type of injury

			%	*N*
Sex	Female		32.9	24
	Male		67.1	49
Nerve	*N. ulnaris*		49.3	36
	*N. radialis*		21.9	16
	*N. medianus*		19.2	14
	Upper and lower plexus		4.1	3
	Upper plexus		2.7	2
	Lower plexus		1.4	1
	*N. musculocutaneus*		1.4	1
Lesioning mechanism	Fracture	Total Humerus Ulna/radius Elbow clavicle	76.7 63.0 8.2 4.1 1.4	56 46 6 3 1
	Cut injury		11.0	8
	Traction		6.8	5
	Iatrogenic		4.1	3
	Contusion		1.4	1
Side	Right		43.8	32
	Left		56.2	41
Treatment	Conservatively		52.1	38
	Operatively		47.9	35
	**Mean**		**Range (min–max)**	***N***
Age (y)	7.0		0–17	73
Time trauma to first presentation	2.89		0–20	73

### Clinical Presentation

**Fig. 1:**
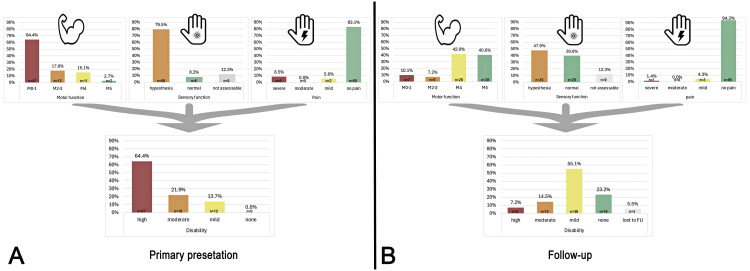
Clinical state of patients on primary presentation ( **A** ) and after a mean follow-up ( **B** ) of 6 months.

At initial presentation, 64.4% ( *n* = 47) of cases exhibited severe motor impairment (M0–M1) or reported severe pain (8.5%, *n* = 6), contributing to a high degree of disability in 64.4% ( *n* = 47) of cases. Clinical details of all nerve injuries at the time of the first consultation are shown in Fig. 1A . No significant correlation was found between age, sex, or the specific injured nerve and the initial level of disability.

### Electrophysiological Results

EMG and/or neurography was performed in 35 patients (47.9%) on initial presentation to our clinic ( Table 2 ). The mean age of patients who underwent any electrophysiological evaluations was 9.6 years (range: 2–17 years). For those receiving EMG and neurography, the mean ages were 11.0 years (range: 5–17 years) and 9.0 years (range: 2–15 years), respectively. Age was significantly associated with the likelihood of undergoing electrophysiological examinations ( *t* (71) = 3.085, confidence interval, CI [1.0–4.6], *p* = 0.003).

**Table 2:** Diagnostic findings of high-resolution nerve ultrasound (HRUS) and electrophysiology on primary presentation

	Diagnostic modality	Findings		%	N
**Primary presentation**	**HRUS**	**Total**		**67.1**	**49**
		*Loss of continuity*		2.0	1
		*Neuroma*		6.1	3
		*Penetration*	*Metal*	2.0	1
			*Bone*	2.0	1
		*Compression*	*Scar*	14.3	7
			*Metal*	10.2	5
			*Bone*	4.1	2
		*Swelling and hematoma*		2.0	1
		*Swelling without compression*		38.8	19
		*unremarkabel*		18.4	9
	**EMG**	**Total**		**27.4**	**20**
		*Insertional activity*	*Spontaneous activity*		
		+	−	10	2
		+	+	70	14
		−	+	10	2
		−	−	10	2
	**Neurography**	**Total**		**39.7**	**29**
		*% of normal motor amplitude*	0.0	44.4	12
			0.1–5	40.7	11
			5.1–10	14.8	4
			>10	0.0	0
		*% of normal sensory amplitude*	0.0	100	23
	**EMG + Neurography performed**	**Total**		**17.8**	**13**
**Follow-up**	**HRUS**	**Total**		**30.1**	**22**
		*Swelling*		63.6	14
		*Neuroma*		13.6	3
		*unremarkabel*		22.7	5
	**EMG**	**Total**		**23.3**	**17**
		*Insertional activity*	*Spontaneous activity*		
		+	−	35.3	6
		+	+	52.9	9
		−	+	11.8	2
		−	−	0.0	17
	**Neurography**	**Total**		**15.1**	**11**
		*% of normal motor amplitude*	0.0	20.0	2
			0.1–5	30.0	3
			5.1–10	30.0	3
			10.1–20	20.0	2
			>20	0.0	0
		*% of normal sensory amplitude*	0.0	77.8	2
			0.1–5	11.1	1
			5.1–10	11.1	1
			>10	0.0	0
	**EMG + Neurography performed**	**Total**		**9.6**	**7**

No statistically significant association was found between EMG findings, motor or sensory amplitudes on neurography, and the degree of disability at presentation or follow-up, nor with the choice of treatment (surgical vs. conservative).

### High-Resolution Ultrasound

**Fig. 2:**
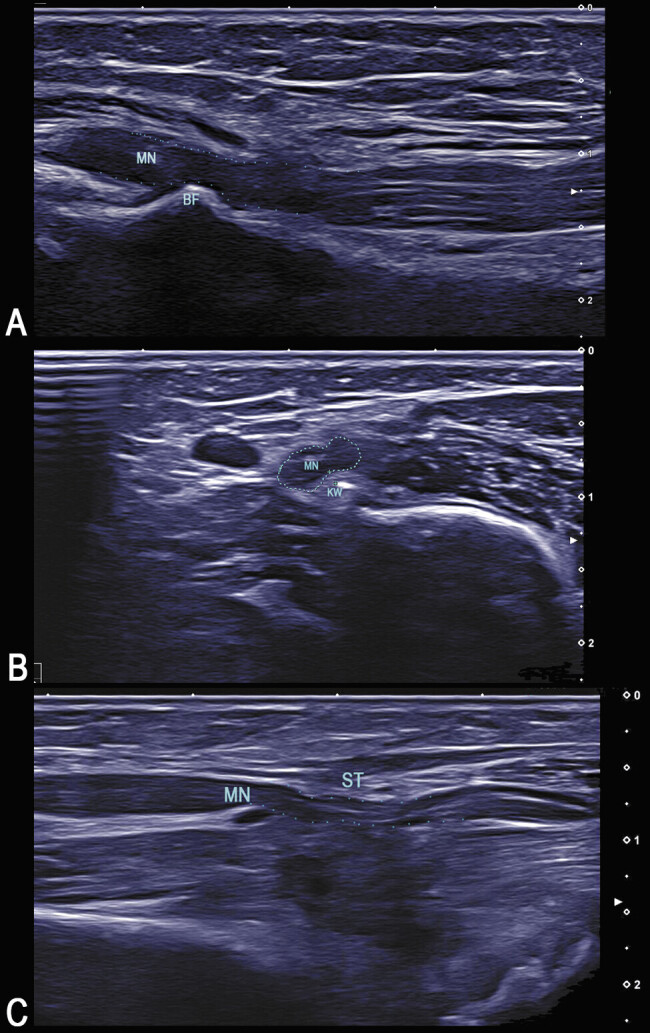
Examples of HRUS findings leading to early surgical exploration. ( **A** ) by a bone fragment (BF). ( **B** ) Compression of the median nerve by a Kirschner wire (KW). ( **C** ) Compression of the median nerve by scar tissue (ST).

**Fig. 3:**
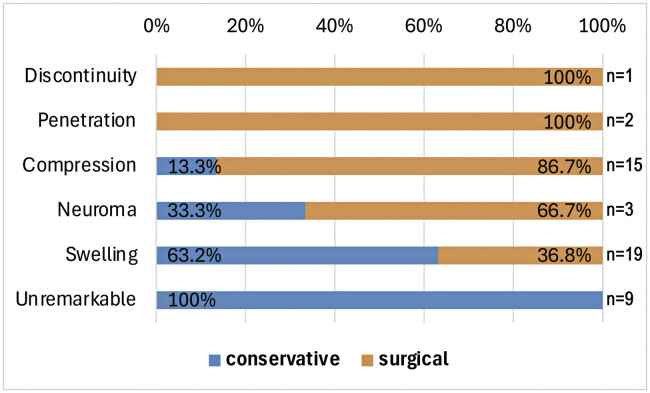
Type of HRUS pathology and consecutive treatment decision (surgical vs. conservative).

HRUS was performed in 49 (67.1%) nerve injuries. The mean age of those who underwent HRUS was 7.8 (SD = 3.9) years with no significant difference compared with the group without HRUS examinations (M = 8.8 ± 4.3, *t* (71) = −1.023, CI [−3.1 to 1.0], *p* = 0.31). All sonographic findings are summarized in Table 2 and examples can be found in Fig. 2 . There was no significant difference in the initial disability grade between patients with and without sonography (χ ^2^ = 0.10, *p* = 0.756). However, HRUS findings were significantly associated with the choice of subsequent treatment (conservative vs. surgical, χ ^2^ = 21.03, *p* < 0.001; Fig. 3 ) and the type of surgery performed in the operative group (χ ^2^ = 4.44, *p* = 0.035).

**Fig. 4:**
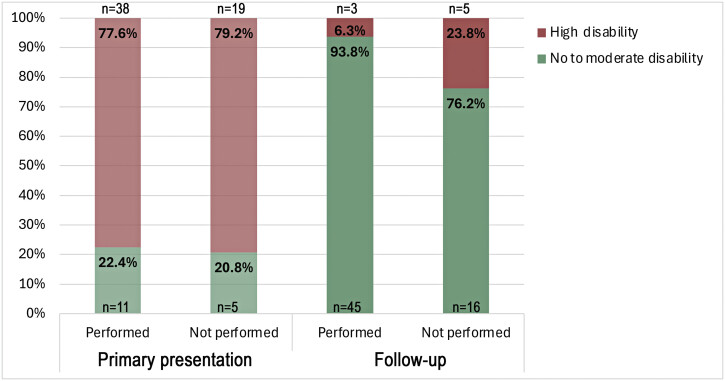
Degree of disability on primary presentation and follow-up of patient groups in whom sonography had been performed or not performed. Rate of high or moderate disability on primary presentation was slightly higher in the group undergoing sonographic diagnostics but not statistically significant (χ ^2^ = 0.10, *p* = 0.756). On follow-up patients with sonography being incorporated in therapeutic decision-making showed significantly higher rates of favorable outcome compared with the group without sonographic assessment (χ ^2^ = 4.26 *, p* = 0.039).

Time to surgery did not differ between patients with or without sonographic evaluation (no HRUS performed: 4.8 ± 3.3 months; HRUS performed: 5.6 ± 5.9 months, U = 150.5, *p* = 0.66). However, initial HRUS was significantly associated with better clinical outcome on follow-up (χ ^2^ = 4.26, *p* = 0.039, Fig. 4 ).

### Treatment of Nerve Injuries

A total of 49.3% ( *n* = 36/73) of injuries were treated surgically, whereas 50.7% ( *n* = 37/73) received conservative treatment. Surgery was performed at a median of 4 months (range: 0–21) posttrauma. An association was found between the initial clinical condition and treatment choice, with a higher rate of surgery in patients with high (55.3%, *n* = 26/47) or moderate (50.0%, *n* = 8/16) compared with those with low disability (20.0%, *n* = 2/10) (χ ^2^ = 3.98, *p* = 0.046). The median follow-up period was the same for both the surgical and conservative groups (6.0 months, Table 3 ). There was no significant difference in clinical outcomes between the surgical and conservative treatment groups, with both showing high rates of no to moderate disability at 6 months follow-up (surgery: 87.5%, *n* = 28/32; conservative: 97.3%, *n* = 36/37, χ ^2^ = 2.45, *p* = 0.12).

**Table 3:** Details of nerve injury treatment

Treatment		% of cases	N
***Surgery***		49.3	36
Surgical procedure	Neurolysis	21.9	16
	Decompression/transposition	8.2	6
	End-to-end-coaptation	2.7	2
	Split-repair (N. suralis)	5.5	4
	Full nerve transplant (N. suralis)	11.0	8
***Conservative***		50.7	37
**Time periods**	**Median [months]**	**Range**	**N**
***Period trauma to surgery***	4.0	0–21	35
***FU period*** Total	6.0	1–89	69
Surgical group	6.5	1–89	32
Conservative group	6.0	1–45	37

### Outcome

**Fig. 5:**
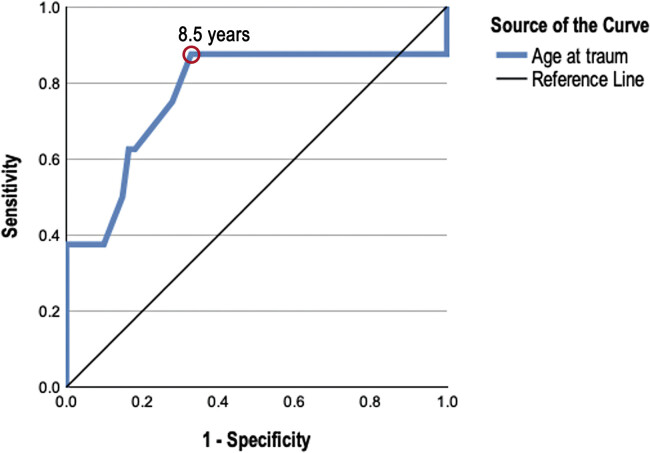
Receiver operating (ROC) curve of age and clinical recovery. Area under the curve = 0.78, the red circle represents the defined cutoff point at 8.5 years with a sensitivity or 80% and a specificity 75%.

Four patients were lost to follow-up and were excluded from further analysis. Overall, 92.8.5% ( *n* = 64/69) of cases showed significant clinical improvement, with full recovery or mild to moderate deficits ( Fig. 1B ). Only one patient (1.4%) revealed no clinical improvement at follow-up. Younger patients also demonstrated significantly better recovery (low or no disability: mean age = 7.4 ± 3.6 years vs. high or moderate disability: mean age = 10.1 ± 5.2 years; *t* (67) = 2.29, CI [0.3, 4.9], *p* = 0.025). Receiver operating curve analysis revealed a cutoff age of 8.5 years (area under the curve = 0.77, sensitivity = 0.88, specificity = 0.67, *p* = 0.015, Fig. 5 ). A significant difference in recovery was observed based on the level of nerve injury, with worse outcomes in cases involving the brachial plexus (χ ^2^ = 9.27, *p* = 0.002). When comparing more peripheral lesions, the best outcomes were observed in ulnar nerve injuries (0.0% with high disability at follow-up, *n* = 0/32), followed by radial nerve injuries (6.3% with high disability at follow-up, *n* = 1/16), whereas the worst outcomes were seen in median nerve injuries (28.6% with high disability at follow-up, *n* = 4/14). Potential age-related bias was excluded. Factors significant in univariable analysis (injured nerve, age, and HRUS) were included in a binary logistic regression model to assess predictors of clinical outcomes at follow-up. The model was significant (χ ^2^ (3) = 17.89, *p* < 0.001), identifying age at the time of trauma (odds ratio [OR] = 1.3, *p* = 0.028), the use of HRUS (OR =10.6, *p* = 0.035), and injured nerve (OR = 3.1, *p* = 0.022) as independent predictors of clinical outcome.

## Discussion

Given the lifelong implications of peripheral nerve lesions in the pediatric population in combination with more complicated circumstances in diagnosing these kinds of injuries in children, there is an urgent need to determine which modalities can facilitate management of such conditions. 4 17 Data on epidemiology and long-term follow ups after surgical or conservative treatment of upper extremity lesions in children are generally sparse. 2 The number of publications addressing this topic has significantly increased in recent years. In the light of ongoing technical innovations in the field of peripheral nerve ultrasound only few authors, however, have investigated if peripheral nerve ultrasound is a useful addition to the armamentarium of physicians involved in the treatment of children with peripheral nerve lesion. Virdee et al. have recently published a meeting abstract of a retrospective study summarizing their 4.5 years of experience with nerve ultrasound in a tertiary referral center. They included 18 pediatric patients of which 11 were treated surgically; therefore, this number corresponds to the percentage of patients who received surgical treatment in our cohort. The authors concluded that nerve ultrasound and nerve conduction studies are valuable tools in this context but advised for larger studies to be undertaken. 17 Further studies in cohorts larger than 18 patients are so far missing in the literature to confirm the additive value of HRUS for a timely diagnosis and management of pediatric peripheral nerve injuries. To our knowledge, no comparative study investigated surgical outcome based on the use of HRUS. This technique offers several advantages, making it particularly, though not exclusively, suitable for examinations in the pediatric age group. First of all, HRUS can be performed regardless of age 11 and requires no sedation or anesthesia as it is commonly the case for MRI scans. 9 10 22 HRUS is also cost-effective and more easily accessible than MRI. It has a much higher diagnostic accuracy compared with standard MRI imaging and is equal to specialized T2 based MR neurography, 23 which is, however, at least in Germany only available in a few specialized centers. Additionally, it allows dynamic examinations to visualize nerve subluxation and gliding. 24 On the other hand, HRUS has the disadvantage of being highly investigator-dependent; therefore, currently limiting the use and accuracy of this examination outside of specialized centers. 22 A further limitation is the accessibility of some nerves, i.e., the proximal sciatic nerve, which can often not be visualized sufficiently by ultrasound. 25 Owing the so far restricted data concerning sonomorphology of nerve traumata, the appearance of the nerve in ultrasound does not automatically allow a conclusion regarding the functionality of the nerve fibers. So, interpretation of ultrasound findings must be done cautiously.

In comparison to electroneuromyographic evaluations that are often painful and require voluntary muscle activation, nerve ultrasound can be performed without the need for invasive measures or voluntary participation of the patient. 14 15 Its diagnostic accuracy has been shown to be equal to electrophysiological examinations in adult cohorts with carpal tunnel syndrome and ulnar neuropathy 26 27 as well as in traumatic nerve injuries. 28 Large studies comparing muscle ultrasound with EMG findings are limited. The two diagnostic methods tend to yield more concordant results in cases of more severe pathologies. 29 However, muscle ultrasound was not a subject of the present study.

Our study demonstrates that there is no age limitation for HRUS, with the youngest patient being under 1 year old. In contrast, EMG was not performed in children younger than 5 years and only conducted with sedation in individuals under 7 years, leading to potentially altered results due to the necessity of voluntary muscle activation.

The results of the presented study showed no significant correlation of EMG and neurographic findings with clinical outcomes. However, it has to be critically considered that the number of examinations performed was limited, and qualitative results are difficult to compare across patients with different lesions, especially in a retrospective setting. This finding therefore is not valid to question the overall reliability of electrophysiological assessments in children but should rather emphasize the challenges of performing these examinations in pediatric patients. As a result, electrophysiology played a secondary role in therapeutic decision-making in the cohort presented.

We found that functional recovery in our patient cohort was age-dependent, marking a threshold of 10 years as cutoff between the group that demonstrated good functional recovery and the group that did not. The influence of age has long since been discussed in literature with contradictory results of studies examining the age dependency of functional recovery following peripheral nerve lesions. 30 31 32 33 34 Several authors report poorer results in patients who had entered the second decade of their life, 35 36 37 which is in accordance with what we found in our patient collective. In a study of sensory function following median or ulnar nerve repair in 54 children, Lundborg et al. stated significantly better recovery in those under the age of 10. They highlight that the ability to reorganize the somatosensory cortex may compensate for axonal misdirection, leading to better outcomes in younger children. This age threshold for neuroplasticity coincides with the critical period of learning a second language as a native speaker. 37 Our work confirms previous reports that highlight the first decade of life as especially favorable in regard to functional recovery following nerve trauma.

Our study is limited by its retrospective character and missing long-term follow-up in several patients. However, in comparison to the literature on this subject we present one of the largest cohorts with a relatively long follow-up period. Nevertheless, we recommend undertaking prospective, multicenter studies to verify the promising results demonstrated in our work.

## Conclusions

In this study regarding children with peripheral nerve injuries of the upper extremities the choice of treatment was mainly determined by thorough clinical examination and the results of the neurosonographic evaluations performed at initial presentation in approximately 68% of injured nerves. Patients younger than 9 years and those in whom HRUS examinations were incorporated in the treatment decision showed better outcomes and the use of HRUS was demonstrated as an independent positive outcome predictor.

While our work, despite a prospective use of ultrasound and entry of patients in the database, was retrospectively analyzed, it is therefore limited in its validity. Nevertheless, it undoubtedly confirms the value of HRUS as a noninvasive tool for diagnosis and management of peripheral nerve injuries, especially in the pediatric population.
